# Maternal and psychosocial antecedents of anxiety and depression in extremely low gestational age newborns at age 15 years

**DOI:** 10.3389/frcha.2024.1334316

**Published:** 2024-09-13

**Authors:** Isha Jalnapurkar, Ali Oran, Jean A. Frazier, David Cochran, Sohye Kim, Elizabeth Jensen, Robert Joseph, Stephen R. Hooper, Hudson Santos, Hernan Jara, Karl C. K. Kuban, Michael E. Msall, Rachana Singh, Lisa Washburn, Semsa Gogcu, Shannon Hanson, Lauren Venuti, Rebecca C. Fry, T. Michael O’Shea

**Affiliations:** ^1^ Eunice Kennedy Shriver Center, University of Massachusetts Chan Medical School, Worcester, MA, United States; ^2^ Department of Pediatrics, University of North Carolina at Chapel Hill, Chapel Hill, NC, United States; ^3^ Epidemiology and Prevention, Wake Forest School of Medicine, Winston-Salem, NC, United States; ^4^ Department of Anatomy & Neurobiology, Boston University Chobanian & Avedisian School of Medicine, Boston, MA, United States; ^5^ University of Miami School of Nursing & Health Studies, Miami, FL, United States; ^6^ Department of Radiology, Boston University Chobanian & Avedisian School of Medicine, Boston, MA, United States; ^7^ Department of Neurology, Boston University Chobanian & Avedisian School of Medicine, Boston, MA, United States; ^8^ Department of Pediatrics, Section of Developmental and Behavioral Pediatrics and Kennedy Research Center on Intellectual and Neurodevelopmental Disabilities, Comer Children's Hospital, Chicago, IL, United States

**Keywords:** adolescents, preterm, anxiety, depression, socioeconomic status, maternal health

## Abstract

**Objectives:**

The prevalence of many psychiatric symptoms, including anxiety and depression, is higher in individuals born extremely preterm (EP) than in term-born individuals during childhood and adolescence. In this prospective study of adolescents born EP, we examined associations between early-life risk factors (prenatal maternal health conditions, socioeconomic and social factors) and anxiety and depression at 15 years of age.

**Methods:**

We included 682 participants (53.2% White, 57.8% male) who were born <28 weeks gestation. Data on demographic factors, maternal health conditions and socioeconomic status (SES) were collected in the first postnatal month, and data on the outcomes (anxiety and depression) were collected at 15 years by a structured clinical diagnostic interview. At the 15-year visit, the mother reported on her own experiences of childhood trauma. Logistic regression models were used to evaluate associations between maternal health indicators, SES factors and mothers' childhood trauma and adolescent outcome variables of anxiety, depression and both anxiety and/or depression, adjusting for potential confounding factors and expressed as adjusted odds ratios (aOR) and 95% confidence intervals (CI).

**Results:**

Maternal pre-pregnancy obesity was associated with anxiety (aOR: 1.84, 95% CI: 1.15, 2.95) and depression (aOR: 1.95, 95% CI: 1.17, 3.23) in adolescents at age 15. Maternal exposure to active or second-hand smoke was associated with depression (aOR: 1.8, 95% CI: 1.08, 3.00) and with anxiety and depression (aOR: 2.83, 95% CI: 1.51, 5.31) at age 15. Other maternal pre-pregnancy health indicators of interest including asthma, hypertension and diabetes mellitus did not demonstrate significant associations with symptoms of anxiety or depression in adolescents at age 15 in univariable and multivariate analyses. Maternal childhood experience of parental upheaval was associated with anxiety and depression (OR: 1.91, 95% CI: 1.01, 3.55) in adolescents, and maternal childhood experience of victim violence was linked with anxiety (OR: 2.4, 95% CI: 1.22, 4.62) and anxiety and depression (OR: 2.49, 95% CI: 1.05, 5.42).

**Conclusion:**

These findings suggest that prenatal maternal health and socioeconomic factors contribute to psychiatric disorders among adolescents born EP. These factors could serve as targets for interventions to improve mental health of individuals born EP.

## 1 Introduction

Nearly 15 million infants worldwide are born preterm [<37 weeks' gestational age (GA)] every year (1). The subgroup of extremely preterm (EP) births (<28 weeks gestation) comprises approximately 6% of all preterm births and less than 1% of all births. Despite advances in technology and care of these infants leading to increased survival, EP and extremely low birth weight (ELBW; <1,000 grams) infants still remain at high risk for death and disability. There remains a 30%–50% mortality and, in survivors, a 20%–50% risk of morbidity (2, 3). The prevalence of psychiatric symptoms during childhood and adolescence is significantly higher in individuals born EP and/or ELBW than in term-born individuals (2–4 times increased odds) (4–8). We have previously reported that at 15 years of age, 11% of girls and 5% of boys born EP have generalized anxiety and 6% of girls and 2% of boys have depression, and these diagnoses are more prevalent when compared to general population epidemiologic adolescent studies in the U.S. (4).

EP infants may undergo altered development of their stress-regulatory systems since the third trimester of pregnancy represents a sensitive phase of infant brain plasticity (9, 10). Due to their immature neurobiological system and subsequent exposure to intensive medical treatments and extended stay in the Neonatal Intensive Care Unit (NICU), the infant's natural regulatory capacity may be exceeded leading to permanent alterations in their neuroendocrine, autonomic, cardiovascular and neuronal responses (11–13). These early exposures to physical and environmental stressors may increase the brain's vulnerability to stress later in life and subsequent development of psychopathology, especially during adolescence (14). EP infants in the NICU may also experience atypical maternal care due to obstruction of physical and emotional closeness, a critical factor in the early regulation of the infant's stress responses, leading to an increased risk of disruption of the maternal-infant attachment (9, 15). Longer NICU hospitalizations have also been associated with worse neurodevelopmental outcomes at 2–3 years of age (16, 17). Despite the higher prevalence of mental health conditions in individuals born EP, few studies have evaluated their mental health outcomes during adolescence.

Anxiety and depressive disorders are among the most important health challenges faced by adolescents in the general population (18). These disorders are common with an estimated lifetime prevalence of 7.3%–28.8% and are associated with substantial functional impairment, with an estimated cost between $42 and $47 billion to the US economy each year (19, 20). Like many other psychiatric conditions, anxiety and depressive disorders have their onset in childhood. Data from the National Comorbidity Survey Replication (NCS-R), a nationally representative epidemiologic study, reported that exposure to early-life stress and trauma has been associated with the development of depression, panic disorder, and an abnormal stress response (21). Results from nationwide birth cohort studies (22, 23), indicated multifactorial etiologies including psychological, social, familial, and biological factors (eg: puberty, hormones and immune system regulation factors) in increasing vulnerability to internalizing disorders like anxiety in adolescent females. The presence of anxiety and depressive symptoms can have significant long-term impacts including poor academic performance, behavioral problems, poor self-worth, and substance use, which may persist into adulthood (24–27). Despite these detrimental outcomes, anxiety disorders in adolescents are undertreated, with only 18% engaged in treatment (28). Given that children born EP are at an increased risk for anxiety and depression during adolescence and young adulthood (4, 26, 29), it is imperative to investigate and identify factors that may contribute to or mitigate the expression of these disorders.

In individuals exposed to significant early-life stress, such as the Extremely Low Gestational Age Newborn (ELGAN) cohort, social environment has an enormous impact on the individual due to its impact on brain development (30). A maternal childhood history of trauma and maltreatment is associated with increased mental health challenges, social isolation, and altered developmental expectations (31). Children of mothers who have had exposure to adverse childhood experiences have an increased risk for anxiety (30), depressive symptoms, aggression, and hyperactivity (32–34), largely due to resultant lack of access to social support and resources that can potentially address these negative exposures (35, 36). Exposure to trauma in childhood can lead to aggressive response biases in adulthood and can thus influence the immediate family environment and caregiving quality in this already vulnerable population (35–37). Thus, in addition to major life events, abuse, and neglect, the day-to-day experiences in family, neighborhood, school, and work environments may affect neurobiological and behavioral functions. Socioeconomic status (SES), which includes both income and education, is also a strong predictor of brain and body health, including anxiety and depressive symptoms (26, 30).

Our objective was to identify early life risk factors that might be associated with EP birth and anxiety and depression later in life in adolescents born EP. This work can provide targets for intervention to ameliorate or prevent anxiety and depression in this population.

## 2 Methods

### 2.1 Study participants

The ELGAN study is a longitudinal, observational study of individuals born EP between 2002 and 2004 in 11 cities in 5 states (38, 39). For the current study, data on the exposure (maternal health conditions and SES) were collected within a few days of the delivery of the ELGAN participant, maternal childhood trauma history and offspring psychiatric outcomes (anxiety and depression) were collected at the age 15-year visit (40). Data about neonatal characteristics were collected from a review of the neonatal medical record at birth. Biological sex was recorded by the neonatologist or pediatrician as biological male, biological female or ambiguous (henceforth referred to as male/female in the manuscript).

During the years 2002–2004, women delivering before 28 weeks' gestation in 11 cities in 5 states were asked to enroll in the first phase of the study. All procedures for this study were approved by the institutional review boards of all participating institutions.

### 2.2 Perinatal data

Data on maternal demographic factors, pre-pregnancy health, pregnancy complications, and medical treatments were collected by maternal interview within a few days of delivery, and by a review of maternal medical records by research assistants, with oversight from neonatologists at each recruitment site. Data included race, ethnicity, marital status, maternal age, health insurance, pre-pregnancy weight and height, active and passive smoke exposure, maternal medical disorders, and maternal medications. Smoke exposure was measured as described in Venkatesh et al., 2021 (41).

Maternal health characteristics included pre-pregnancy body mass index (BMI) categories (underweight: <18.5, healthy weight: 18.5–25, overweight: >25–30, and obese: >30), pre-pregnancy diagnosis of asthma and diabetes, and pre-pregnancy/pregnancy/delivery hypertension symptoms. Potentially life-threatening maternal pregnancy complications and indications for premature delivery, as well as Hemolysis, Elevated Liver enzymes and Low Platelets (HELLP) syndrome and preeclampsia, were included. A composite variable was defined as having displayed any of the aforementioned hypertensive disorders, HELLP, or preeclampsia. Whether mother smoked or was exposed to second-hand smoke during pregnancy was ascertained by maternal interview; and a composite exposure was defined for mothers' who either smoked or were exposed to second-hand smoke during pregnancy.

The following socioeconomic variables were included: education (less than high school), marital status (not married or not living together), insurance (public insurance or no insurance), and Supplemental Nutrition Assistance Program (SNAP) eligibility.

Newborn characteristics included sex, birth weight, gestational age, and medical conditions known to commonly occur in EP infants such as, intraventricular hemorrhage, white matter damage, chronic lung disease, sepsis, severe retinopathy of prematurity, and necrotizing enterocolitis. Since the focus of the current manuscript was on maternal health and psychosocial antecedents of anxiety and depression, these variables were not included and reported in the present analysis.

The Childhood Trauma Questionnaire [CTQ; (42); ECHO-wide Cohort version 01.20] was completed by 438 mothers during the age 15 visit. It provided a description of maternal exposure to death of a family member/very close friend, parent upheaval such as divorce, separation, etc., sexual trauma, victim violence (other than sexual violence), being extremely ill/injured, or any other upheaval. It also obtained data on a 7-point Likert scale on how traumatic the experience was for the mother. Internal consistency in a community sample was acceptable for the entire measure (Cronbach's alpha = 0.91) and four of the five subscales (ranging from 0.58 for Physical Neglect to 0.94 for Sexual Abuse (43). In clinical samples, the CTQ has demonstrated test-retest reliability coefficients from 0.79 to 0.86, internal consistency reliability coefficients ranging from 0.66 to 0.92, convergent validity with clinician ratings of abuse, and a consistent five-factor structure (42).

### 2.3 Neuropsychiatric assessments

The Mini International Neuropsychiatric Interview for Children and Adolescents (MINI-KID 7.0.2) (44) is a structured clinical diagnostic interview tool designed to assess the presence of current DSM-5 and ICD-10 psychiatric disorders in children and adolescents aged 6–17 years. It is used for psychiatric evaluation and outcome monitoring in clinical psychopharmacology trials and epidemiological studies in more than 100 countries. The interview is administered to the child/adolescent together with the parent(s), although it can be administered to adolescents without a parent present. The MINI-KID follows the structure and format of the adult version of the interview (MINI), which has been validated against the Structured Clinical Interview for DSM-III-R and against the World Health Organization–designed Composite International Diagnostic Interview. Like its adult counterpart, the MINI-KID is organized in diagnostic sections or modules, and is administered using branching tree logic (e.g., 2–4 screening questions for each disorder, with additional questions being asked only if the screen questions are positively endorsed). The instrument screens for 24 DSM-5 and ICD-10 psychiatric disorders and suicidality and takes approximately 30 min to complete. The MINI-KID has substantial to excellent concordance with the gold standard K-SADS-PL (area under the curve = 0.81–0.96, ≥0.56–0.87). Sensitivity was 0.61–1.00 for 15/20 individual disorders, and specificity was 0.81–1.00 for 18 disorders and >0.73 for the remaining two. Interrater and test-retest kappas were 0.64–1.00 for all individual disorders except dysthymia. It has recently been updated to map onto DSM-5 (MINI 7.0.2) diagnostic criteria.

The primary outcomes for our analyses were presence of any form of anxiety (i.e., generalized anxiety disorder, separation anxiety disorder, social anxiety disorder, panic disorder lifetime/current, specific phobia, and agoraphobia), depression (major depressive disorder current/recurrent/past), as assessed by the MINI-KID, anxiety-or-depression, in which participants met the cut-offs for either disorder, and anxiety-and-depression, indicating that they met cut-offs for the comorbidity of both disorders, thereby indicating greater illness severity.

### 2.4 Statistical analysis

We evaluated the association between the following sets of exposures: maternal health conditions, SES indicators during pregnancy, history of maternal childhood trauma with four outcomes: anxiety, depression, and anxiety-or-depression, and anxiety-and-depression at 15 years of age. Based on sex differences in the prevalence of psychiatric disorders, we also evaluated sex as a modifier of associations between perinatal factors and adolescent psychiatric disorder (45, 46). Regression models were used to evaluate associations between psychiatric outcomes of anxiety, depression, anxiety-or-depression, and anxiety-and-depression, and antecedents, including prenatal maternal health factors as well as SES factors. For maternal education and marital status variables, both of which consisted of five categories, a secondary set of binary variables- college education and married- were also defined to explore these factors' associations without the small sample size effects of some categories. In the same regard, the very small number of mothers whose marital status were “widowed” were analyzed along with mothers “separated or divorced”, under the “separated or divorced or widowed” category (Table 2). Statistical significance was defined as *p* < 0.05. After conducting univariate analyses, multivariate regression analyses were conducted. The variables for adjustment were determined using directed acyclic graphs, DAGs, to determine a minimally sufficient set of adjustment variables for inclusion in the models (see Supplementary Figures). Adjustment variables are listed in Supplementary Table 1. Based on the finding that pre-pregnancy exposure to adverse experiences has been associated with increased risk of maternal health conditions like hypertension, preterm birth and detrimental maternal mental health outcomes (31), severity of trauma as measured by the CTQ was included as a confounding variable.

## 3 Results

**Figure 1:**
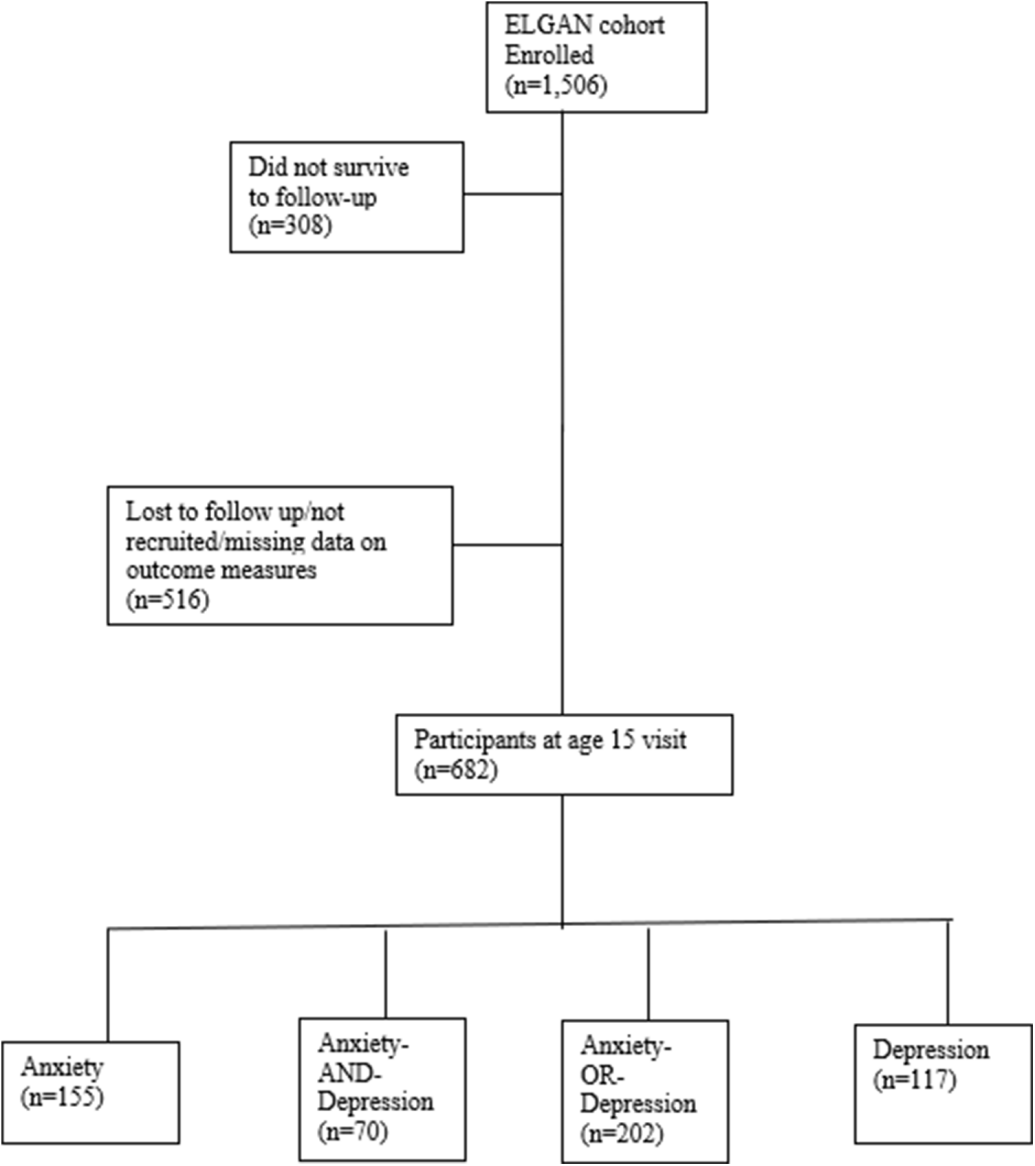


A total of 1,506 infants, born to 1,249 mothers, were enrolled in the ELGAN study. Of the 1,198 children assessed in this longitudinal study at age ten, 516 participants were lost to follow-up or not recruited in this age 15 analysis. Four participants did not have any measures of depression or anxiety completed and were excluded from this current report. Youth with anxiety symptoms (*N* = 155) included those with anxiety alone (*N* = 85) and those with anxiety and depression comorbid (*N* = 70). Youth under the depression category (*N* = 117) included those with depression only (*N* = 47) and those with both, depression and anxiety symptoms (*N* = 70; see Figure 1). No significant differences were noted in demographic characteristics, maternal health and socioeconomic variables among those assessed and not assessed at age 15 (see Table 1). Given that anxiety and depressive disorders are highly comorbid with a significant overlap in symptoms, this combination can contribute to an increased risk of comorbid general medical illnesses, and worse treatment outcomes than with either condition alone (47–50), the combined variable, anxiety-and-depression, was an indicator of greater illness burden in our analyses at age 15 in the ELGAN cohort.

**Table 1:** Characteristics of ELGAN study participants.

Characteristic	Died by age 10	Alive not in sample	Alive in sample
	*N* = 308	*N* = 516	*N* = 682
Maternal characteristics			
Maternal age			
Under-21	48 (15.7%)	89 (17.2%)	81 (11.9%)
21–35	215 (70.3%)	350 (67.8%)	452 (66.3%)
Over-35	43 (14.1%)	77 (14.9%)	149 (21.8%)
(missing)	2	0	0
Race			
White	160 (53.2%)	257 (50.8%)	449 (66.7%)
Black	105 (34.9%)	165 (32.6%)	157 (23.3%)
Asian	6 (2.0%)	15 (3.0%)	13 (1.9%)
Native American	2 (0.7%)	10 (2.0%)	2 (0.3%)
Mixed Race	14 (4.7%)	16 (3.2%)	19 (2.8%)
Other	14 (4.7%)	43 (8.5%)	33 (4.9%)
(missing)	7	10	9
Hispanic Ethnicity	32 (10.7%)	84 (16.4%)	63 (9.3%)
(missing)	8	3	3
Marital status			
Married	163 (53.6%)	242 (46.9%)	443 (65.1%)
Separated or divorced	9 (3.0%)	15 (2.9%)	26 (3.8%)
Not ever married but living together	61 (20.1%)	133 (25.8%)	112 (16.4%)
Not ever married and not living together	70 (23.0%)	126 (24.4%)	99 (14.5%)
Widowed	1 (0.3%)	0 (0.0%)	1 (0.1%)
(missing)	4	0	1
Education			
Less than high school	50 (20.1%)	107 (21.9%)	85 (12.8%)
High school graduate	77 (30.9%)	155 (31.7%)	159 (24.0%)
Some College	70 (28.1%)	117 (23.9%)	153 (23.1%)
College Graduate	35 (14.1%)	66 (13.5%)	147 (22.2%)
More than College	17 (6.8%)	44 (9.0%)	119 (17.9%)
(missing)	59	27	19
Eligible forMedicaid	130 (49.6%)	243 (48.4%)	221 (32.9%)
(missing)	46	14	11
Eligible for Food Stamps	49 (18.9%)	92 (18.4%)	74 (11.0%)
(missing)	49	16	11
Maternal health characteristics			
Body mass index	27 (7.9)	25 (6.3)	26 (6.9)
(missing)	57	26	25
Prepregnancy body mass index			
Healthy Weight	96 (38.2%)	254 (51.8%)	329 (50.1%)
Under Weight	18 (7.2%)	42 (8.6%)	48 (7.3%)
Over Weight	62 (24.7%)	99 (20.2%)	127 (19.3%)
Obese	75 (29.9%)	95 (19.4%)	153 (23.3%)
(missing)	57	26	25
Prepregnacyasthma	44 (17.1%)	68 (13.7%)	88 (13.2%)
(missing)	50	18	16
Prepregnacy diabetes	9 (3.5%)	13 (2.6%)	21 (3.2%)
(missing)	50	18	16
Prepregnancy high blood pressure	24 (9.3%)	41 (8.2%)	41 (6.2%)
(missing)	50	18	16
hypertension during pregnancy	50 (19.5%)	77 (15.5%)	100 (15.0%)
(missing)	51	19	17
Preeclampsia	25 (9.7%)	46 (9.3%)	68 (10.2%)
(missing)	51	19	17
Preeclampsia or pmom dschg dx pih/tox/eclamp ob1	39 (12.7%)	72 (14.0%)	96 (14.1%)
Mom dschg dx hellp ob1	14 (4.5%)	14 (2.7%)	30 (4.4%)
Hypertension prepregnany or during pregnancy	69 (22.4%)	103 (20.0%)	140 (20.5%)
Smoked while pregnant	50 (19.5%)	69 (13.8%)	93 (13.9%)
(missing)	51	17	14
Passive smoke exposure	75 (29.8%)	142 (28.7%)	151 (22.7%)
(missing)	56	21	16
Smoked while pregnant or prenatal active passive Smoke Exposure	94 (36.6%)	162 (32.5%)	176 (26.3%)
(missing)	51	17	14
Newborns characteristics			
Sex-Male	178 (57.8%)	270 (52.3%)	351 (51.5%)
Birth Weight *Z*-Score			
<−2	48 (15.6%)	20 (3.9%)	42 (6.2%)
<−1	58 (18.8%)	67 (13.0%)	86 (12.6%)
−1 to 1	189 (61.4%)	371 (71.9%)	469 (68.8%)
>1	12 (3.9%)	50 (9.7%)	71 (10.4%)
>2	1 (0.3%)	8 (1.6%)	14 (2.1%)
Gestational Age			
23–24	164 (53.2%)	100 (19.4%)	145 (21.3%)
25–26	108 (35.1%)	241 (46.7%)	312 (45.7%)
27	36 (11.7%)	175 (33.9%)	225 (33.0%)
Intraventricular hemorrhage	107 (41.3%)	88 (17.1%)	156 (22.9%)
(missing)	49	2	0
White matter damage	105 (40.5%)	103 (20.0%)	139 (20.4%)
(missing)	49	2	0
Chronic lung disease	46 (86.8%)	237 (46.5%)	361 (53.2%)
(missing)	255	6	4
Sepsis			
None	105 (43.4%)	189 (36.7%)	256 (37.5%)
Presumed	59 (24.4%)	177 (34.4%)	233 (34.2%)
Sepsis	78 (32.2%)	149 (28.9%)	193 (28.3%)
(missing)	66	1	0
Severe retinopathy of prematurity	17 (23.9%)	61 (12.1%)	95 (14.1%)
(missing)	237	11	10
Necrotizing enterocolitis			
None or mild	244 (80.3%)	479 (92.8%)	631 (92.5%)
Medical necrotizing enterocolitis	5 (1.6%)	6 (1.2%)	5 (0.7%)
Surgical necrotizing enterocolitis	38 (12.5%)	15 (2.9%)	27 (4.0%)
Intestinal perforation	17 (5.6%)	16 (3.1%)	19 (2.8%)
(missing)	4	0	0

### 3.1 Maternal health characteristics at birth

#### 3.1.1 Univariate analyses

Maternal hypertension was significantly associated with symptoms of anxiety and depression in youth at age 15. Specifically, hypertensive disorder during pregnancy was linked with depression (OR: 1.59, 95% CI: 1.00, 2.50). A discharge diagnosis of hypertensive disorder in the mother was associated with anxiety (OR: 1.68, 95% CI: 1.03, 2.67), which was noted in females (OR: 1.81, 95% CI: 1.03, 3.15) but not males. Another maternal health characteristic of significance was pre-pregnancy BMI. In female participants, maternal obesity displayed significant relationships with anxiety (OR: 1.86, 95% 95% CI: 1.03, 3.38), depression (OR: 2.57, 95% CI: 1.33, 5.02), anxiety-or-depression (OR 1.95, 95% CI 1.10, 3.46), and anxiety-and-depression variables (OR 2.9, 95% CI 1.36, 3.61). Maternal smoking during pregnancy was associated with depression (OR 1.73, 95% CI 1.01, 2.87), and anxiety-and-depression (OR: 2.00, 95% CI: 1.06, 3.61). Maternal exposure to second-hand smoke was also associated with depression (OR: 1.73, 95% CI: 1.10, 2.68) and anxiety-and-depression (OR: 1.79, 95% CI: 1.03, 3.03) in the overall sample. This was also noted in females whose mothers were exposed to secondhand smoke with presence of depression (OR: 2.26, 95% CI: 1.26, 4.00) and anxiety-and-depression (OR: 2.76, 95% CI: 1.45, 5.24; see Table 2 and Supplementary Table 2A and 2B for sex differences in outcome variables).

**Table 2:** Maternal health characteristics at birth: univariate and multivariate associations with adolescent anxiety, depression, anxiety-or-depression, and anxiety-and-depression.

Characteristic	Anxiety		Depression		Anx. OR Dep.		Anx. AND Dep.	
	OR	95% CI	OR	95% CI	OR	95% CI	OR	95% CI
Pre-pregnancy asthma	1.13	0.66, 1.87	1.29	0.72, 2.22	1.04	0.63, 1.68	1.62	0.81, 3.01
Pre-preg diabetes	1.34	0.47, 3.37	1.54	0.49, 4.02	1.46	0.57, 3.53	1.46	0.34, 4.46
Pre-preg high blood pressure	1.08	0.49, 2.18	1.39	0.61, 2.89	1.23	0.62, 2.37	1.22	0.41, 2.95
Pregnancy-induced hypertension	1.28	0.78, 2.05	1.16	0.66, 1.97	1.25	0.79, 1.95	1.22	0.60, 2.28
Preeclampsia	1.56	0.88, 2.67	1.16	0.59, 2.14	1.41	0.83, 2.37	1.37	0.61, 2.77
Discharge diagnosis of Hypertensive disorder	**1**.**68**	**1.03, 2.67**	1.65	0.97, 2.73	**1**.**69**	**1.07, 2.63**	1.79	0.94, 3.24
HELLP syndrome	1.04	0.40, 2.35	2.16	0.92, 4.71	1.4	0.63, 2.95	1.81	0.59, 4.52
Hypertensive disorder during pregnancy	1.49	0.97, 2.25	**1**.**59**	**1.00, 2.50**	**1**.**54**	**1.04, 2.28**	1.64	0.92, 2.82
Prepreg BMI								
Healthy Weight	—	—	—	—	—	—	—	—
Under Weight	1.49	0.71, 2.97	1.26	0.52, 2.75	1.61	0.82, 3.05	1.02	0.29, 2.76
Over Weight	**1**.**84**	**1.14, 2.95**	1.55	0.89, 2.63	**1**.**71**	**1.09, 2.66**	1.85	0.96, 3.46
Obese	**1**.**87**	**1.19, 2.92**	**1**.**94**	**1.19, 3.16**	**2**.**08**	**1.37, 3.14**	1.78	0.96, 3.25
Smoke while pregnant	1.43	0.86, 2.31	**1**.**73**	**1.01, 2.87**	1.42	0.89, 2.24	**2**.**00**	**1.06, 3.61**
Second-hand smoke exposure	1.10	0.72, 1.68	**1**.**73**	**1.10, 2.68**	1.22	0.82, 1.79	**1**.**79**	**1.03, 3.03**
Active or Second-hand Smoke Exposure	1.19	0.79, 1.77	**1**.**8**	**1.17, 2.75**	1.26	0.86, 1.81	**2**.**03**	**1.20, 3.37**
Multivariate analyses								
Pre-pregnancy asthma Adjusted for Medicaid, Maternal education	1.08	0.62, 1.81	1.14	0.62, 1.99	0.98	0.58, 1.60	1.41	0.69, 2.70
Pre-pregnancy diabetes Adjusted for Medicaid, maternal education, maternal BMI	1.06	0.36, 2.72	1.18	0.37, 3.20	1.1	0.42, 2.72	1.17	0.26, 3.73
Maternal hypertension combined variable Adjusted for Medicaid, maternal education, maternal BMI, maternal smoke exposure	1.57	0.97, 2.51	1.57	0.97, 2.51	1.46	0.96, 2.20	1.51	0.83, 2.66
Prepreg Body Mass Index: Adjusted for Medicaid, maternal education								
Healthy Weight	—	—	—	—	—	—	—	—
Under Weight	1.46	0.69, 2.93	1.2	0.49, 2.64	1.51	0.76, 2.90	1.04	0.29, 2.85
Over Weight	**1**.**82**	**1.12, 2.95**	1.49	0.85, 2.55	**1**.**71**	**1.08, 2.68**	1.71	0.88, 3.26
Obese	**1**.**84**	**1.15, 2.94**	**1**.**95**	**1.17, 3.23**	**2**.**08**	**1.35, 3.20**	**1**.**77**	**0.94, 3.32**
Active or Second-hand Smoke Exposure	1.11	0.68, 1.78	**1**.**8**	**1.08, 3.00**	1.03	0.66, 1.58	**2**.**83**	**1.51, 5.31**

OR, odds ratio, CI, confidence interval; HELLP, Hemolysis, Elevated Liver enzymes and Low Platelets.

Variables adjusted for analyses: pre-pregnancy asthma, pre-pregnancy diabetes, maternal hypertension adjusted variable, pre-pregnancy body mass index, active or second-hand smoke exposure. Statistically significant associations are in bold.

#### 3.1.2 Multivariable analyses

Mothers who were overweight (BMI: 25.0–29.9) had youth with higher odds of anxiety (aOR: 1.82, 95% CI: 1.12, 2.95) when adjusted for insurance/Medicaid and maternal education. Mothers who were classified as being obese (BMI > 30) had youth with higher odds of anxiety (aOR: 1.84, 95% CI: 1.15, 2.94), depression (aOR: 1.95, 95% CI: 1.17, 3.23), and anxiety-and-depression (aOR: 1.77, 95% CI: 0.94, 3.32). Female youth who had mothers with BMI > 30 were more likely to have depression (OR: 2.44, 95% CI: 1.23, 4.88) and anxiety-and-depression (aOR: 2.58, 95% CI: 1.17, 5.82) when adjusted for Medicaid and maternal education. In male youth, anxiety symptoms were associated with overweight status in mothers (aOR: 2.27, 95% CI: 1.07, 4.75) when adjusted for Medicaid and maternal education. When adjusted for Medicaid status, maternal education and marital status of the mother, smoke exposure (active and second-hand smoke exposure combined) was associated with depression (aOR: 1.8, 95% CI: 1.08, 3.00) and anxiety-and-depression (aOR: 2.83, 95% CI: 1.51, 5.31).

### 3.2 Maternal socioeconomic characteristics

#### 3.2.1 Univariate analyses

Youth whose mother had a high school education were more likely to have anxiety-and-depression (OR: 2.46, 95% CI: 1.03, 6.84) than those with less than college education or higher with stronger associations noted in females (OR: 6.27, 95% CI: 1.70, 40.6). Mothers who did not have a college degree were also more likely to have female children with depression (OR: 1.86, 95% CI: 1.04, 3.45) and anxiety-and-depression (OR: 2.26, 95% CI: 1.13,4.82). Female youth whose mothers were on Medicaid displayed greater likelihood for the development of anxiety (OR: 1.71, 95% CI: 1.04, 2.81) while males had decreased likelihood for the presence of anxiety-and-depression (OR: 0.19, 95% CI: 0.03, 0.67; see Table 3 and Supplementary Table 3A and 3B for sex differences in outcome variables).

**Table 3:** Maternal socioeconomic characteristics at birth: univariate and multivariable associations with adolescent anxiety, depression, anxiety-or-depression, and anxiety-and-depression.

Characteristic	Anxiety		Depression		Anx. OR Dep.		Anx. AND Dep.	
	OR	95% CI	OR	95% CI	OR	95% CI	OR	95% CI
Marital status								
Married	—	—	—	—	—	—	—	—
Separated/Divorced/Widowed	1.97	0.84, 4.36	1.5	0.53, 3.63	1.81	0.80, 3.97	1.75	0.57, 4.48
Never married, living together	0.64	0.36, 1.09	1.35	0.79, 2.26	1.2	0.76, 1.87	0.44	0.16, 0.97
Never married, not living together	1.13	0.67, 1.85	1.01	0.54, 1.79	1.32	0.82, 2.09	0.68	0.29, 1.40
Married	1.05	0.72, 1.54	0.8	0.53, 1.21	0.76	0.54, 1.06	1.51	0.88, 2.68
Education								
Less than High School	—	—	—	—	—	—	—	—
High School	1.2	0.66, 2.25	1.26	0.66, 2.49	0.94	0.54, 1.65	**2**.**46**	**1.03, 6.84**
Less than college	0.89	0.48, 1.70	0.76	0.38, 1.56	0.67	0.38, 1.19	1.43	0.56, 4.15
College	0.87	0.46, 1.66	0.84	0.42, 1.72	0.73	0.41, 1.30	1.28	0.48, 3.76
More than college	0.91	0.47, 1.78	0.67	0.31, 1.44	0.65	0.35, 1.18	1.34	0.49, 4.03
Without College Degree	1.17	0.81, 1.70	1.32	0.87, 2.02	1.21	0.86, 1.72	1.32	0.79, 2.25
Insurance: Medicaid	1.25	0.85, 1.81	1.27	0.83, 1.92	**1**.**47**	**1.04, 2.07**	0.93	0.53, 1.56
Support: Food Stamps	0.99	0.54, 1.73	1.39	0.74, 2.46	1.23	0.73, 2.04	1.05	0.45, 2.16
*Multivariable analyses*								
Marital Status Adjusted for maternal age								
Separated/Divorced/Widowed	1.97	0.84, 4.36	1.5	0.53, 3.63	1.81	0.80, 3.97	1.75	0.57, 4.48
Never married, living together	0.64	0.36, 1.09	1.35	0.79, 2.26	1.2	0.76, 1.87	0.44	0.16, 0.97
Never married, not living together	1.13	0.67, 1.85	1.01	0.54, 1.79	1.32	0.82, 2.09	0.68	0.29, 1.40
Married	—	—	—	—	—	—	—	—
Education Adjusted for maternal age								
Less than High School	—	—	—	—	—	—	—	—
High School	1.16	0.62, 2.20	1.22	0.63, 2.46	0.93	0.53, 1.64	2.26	0.92, 6.40
Less than college	0.84	0.43, 1.66	0.72	0.35, 1.55	0.65	0.35, 1.20	1.25	0.46, 3.77
College	0.8	0.40, 1.61	0.78	0.37, 1.69	0.7	0.38, 1.32	1.07	0.38, 3.33
More than college	0.83	0.40, 1.72	0.61	0.27, 1.40	0.61	0.32, 1.19	1.11	0.38, 3.53
Insurance: Medicaid Adjusted for marital status, maternal age, maternal education	1.3	0.76, 2.23	1.1	0.61, 1.99	1.33	0.81, 2.17	1.03	0.49, 2.13

OR, odds ratio; CI, confidence interval.

Statistically significant associations are in bold.

#### 3.2.2 Multivariable analyses

When adjusted for maternal age, no statistically significant associations were noted between maternal marital status, maternal education and outcomes of anxiety and/or depression in the overall sample as well as in females and males. When adjusted for marital status, maternal age and maternal education, there were no significant associations noted with Medicaid/insurance status.

### 3.3 Maternal childhood trauma history

#### 3.3.1 Univariable analyses

Mother's childhood experience of parental upheaval, defined as experiencing parental separation or divorce, was associated with anxiety-and-depression (OR: 1.91, 95% CI: 1.01, 3.55) in both male and female adolescents, and was associated with anxiety among male adolescents (OR: 2.26, 95% CI: 1.08, 4.65). Maternal childhood exposure to sexual trauma was associated with anxiety-and-depression in female adolescents (OR: 2.55, 95% CI: 1.12, 5.67). Mothers who were victims of violence in childhood were more likely to have adolescents with anxiety (OR: 2.4, 95% CI: 1.22, 4.62), and anxiety-and-depression (OR: 2.49, 95% CI: 1.05, 5.42). In the analysis by sex, this association was only seen in the male offspring. Other upheaval, defined as any other experience or upheaval that may have shaped the reporter's life or personality significantly, was associated with anxiety-and-depression in the overall sample (OR: 2.21, 95% CI: 1.04, 4.44), and anxiety (OR: 3.21, 95% CI: 1.30, 7.68) and anxiety-and-depression (OR: 5.83, 95% CI: 1.80, 18.0) in males. See Table 4 describing maternal childhood trauma characteristics with univariate associations with adolescent anxiety or depression with overall sample, female and male participants.

**Table 4:** Maternal childhood trauma characteristics: univariate associations with adolescent anxiety or depression with overall sample, female and male participants.

Characteristic	Anxiety		Depression		Anx. OR Dep.		Anx. AND Dep.	
	OR	95% CI	OR	95% CI	OR	95% CI	OR	95% CI
Overall sample								
Death of friend/family	0.87	0.55, 1.37	1.16	0.70, 1.91	1.1	0.73, 1.67	0.78	0.41, 1.44
Parent Upheaval	1.5	0.93, 2.42	1.48	0.86, 2.51	1.37	0.87, 2.14	**1**.**91**	**1.01, 3.55**
Sexual trauma	1.6	0.93, 2.70	1.66	0.92, 2.93	1.57	0.95, 2.58	1.91	0.94, 3.69
Victim Violence	**2**.**4**	**1.22, 4.62**	1.78	0.82, 3.63	**2**.**04**	**1.06, 3.89**	**2**.**49**	**1.05, 5.42**
Illness/Injury	0.69	0.25, 1.62	0.83	0.27, 2.05	0.59	0.23, 1.33	1.13	0.32, 3.03
Other Upheaval	1.46	0.80, 2.61	1.76	0.91, 3.26	1.38	0.78, 2.40	**2**.**21**	**1.04, 4.44**
Female								
Death of friend/family	1.04	0.57, 1.88	0.84	0.42, 1.65	1.06	0.60, 1.87	0.77	0.35, 1.65
Parent Upheaval	1.08	0.56, 2.03	1.77	0.87, 3.53	1.16	0.62, 2.12	1.8	0.81, 3.88
Sexual trauma	1.56	0.78, 3.08	2.05	0.96, 4.26	1.45	0.73, 2.80	**2**.**55**	**1.12, 5.67**
Victim Violence	1.62	0.61, 4.06	1.72	0.58, 4.55	1.55	0.60, 3.84	1.96	0.60, 5.47
Illness/Injury	0.51	0.12, 1.64	0.86	0.19, 2.78	0.4	0.09, 1.27	1.26	0.28, 4.17
Other Upheaval	0.77	0.32, 1.69	1.38	0.57, 3.10	0.94	0.43, 1.96	1.15	0.40, 2.86
Male								
Death of friend/family	0.7	0.34, 1.40	1.74	0.82, 3.78	1.17	0.63, 2.15	0.82	0.27, 2.36
Parent Upheaval	**2**.**26**	**1.08, 4.65**	1.13	0.47, 2.55	1.64	0.83, 3.17	2.00	0.64, 5.83
Sexual trauma	1.54	0.63, 3.50	1.14	0.40, 2.85	1.67	0.76, 3.54	0.73	0.11, 2.80
Victim Violence	**3**.**82**	**1.45, 9.83**	1.88	0.58, 5.25	**2**.**75**	**1.08, 6.92**	**3**.**81**	**0.97, 12.6**
Illness/Injury	0.98	0.22, 3.23	0.78	0.12, 2.99	0.89	0.24, 2.67	0.86	0.05, 4.74
Other Upheaval	**3**.**21**	**1.30, 7.68**	2.34	0.84, 5.95	2.2	0.92, 5.11	**5**.**83**	**1.80, 18.0**

#### 3.3.2 Multivariable analyses with severity of traumatic experiences

When adjusted for maternal age and CTQ severity rating, mothers who were exposed to smoke (active and secondhand smoke exposure) had a greater likelihood of having children with anxiety-and-depression (OR: 3.26, 95% CI: 1.39, 7.63). When adjusting for CTQ severity, mothers whose marital status was classified as not ever married, living together, had a greater likelihood of having children with anxiety-and-depression at age 15 years (aOR: 029, 95% CI: 0.07, 0.85).

## 4 Discussion

Children born extremely preterm are at a higher risk of psychiatric disorders in adolescence, notably anxiety and depression (4, 26, 51). We sought to identify modifiable prenatal and early-life risk factors of these highly prevalent conditions in this already vulnerable population by examining associations between youth's psychiatric diagnoses of anxiety and depression and measures of maternal health, socioeconomic factors, and maternal report of childhood trauma in this study. Our study assessed for sex differences for anxiety and depression in the youth born extremely preterm and noted the findings are consistent with sex differences in the prevalence of psychiatric disorders reported in adolescents in epidemiologic studies in the general population (4, 52, 53).

We noted that maternal health attributes of hypertension, elevated BMI (overweight or obese status), and smoke exposure (active or secondhand smoke) were associated with the presence of anxiety and depression in the youth. After adjusting for maternal education and Medicaid status, elevated maternal BMI continued to be significantly associated with anxiety and depression in adolescents. In contrast to earlier findings from our group that did not find an association between smoke exposure and cognitive outcomes at age 10 (41), we noted that smoke exposure even after statistical adjustment for Medicaid, maternal education and marital status remained associated with psychiatric outcomes at age 15. Exposure to maternal metabolic disorders during pregnancy, including hypertension and obesity, has been linked to adverse outcomes in the behavior and physiology of their offspring, and the development of neuropsychiatric disorders such as anxiety and depression (54–57). High maternal BMI increases the risk of adverse childhood outcomes including preterm and extreme preterm birth as well as likelihood of high or low birth weight (58, 59), which in turn increases the risk of the development of anxiety and depression in adolescence (4). A comprehensive meta-analysis by Zhang and colleagues also demonstrated associations between maternal pre-pregnancy overweight status/obesity and adverse neurodevelopmental outcomes in their offspring including anxiety, depression, emotional symptoms, autism spectrum disorder among others. Obesity during pregnancy can have an impact on neuroendocrine, metabolic and inflammatory systems leading to altered neuronal plasticity, impaired reward circuitry, and dysregulated brain metabolism (60–62). Additionally, due to disturbances in metabolic and endocrine factors in adipose tissue in obesity, there is an increased risk of insulin resistance which can increase the risk for the development of type 2 diabetes (63, 64). A nationwide cohort study in Finland (65) found that maternal moderate and severe obesity along with type 2 diabetes and pregestational diabetes were associated with development of mood disorders in their children. The association of severe obesity in combination with diabetes had a stronger link with psychiatric and neurodevelopmental outcomes in this study, suggesting a stronger neural exposure to inflammation, oxidative stress, lipotoxicity and insulin resistance. In the Avon Longitudinal Study for Parents and Children (ALSPAC), hypertensive pregnancy disorders in mothers predicted a significantly greater risk of anxiety and depression in children at age 7 and age 15 respectively (66). In another large cohort study, maternal smoking in early pregnancy was associated with childhood internalizing symptoms of anxiety and depression (67). It has been hypothesized based on animal models, that exposure to nicotine and other components of cigarette smoke may interfere with neurodevelopmental processes *in utero*, thus leading to this increased risk (68, 69); however, no direct causal relationship between these factors has been demonstrated in human studies (67).

The link between SES and child mental health and wellbeing is well established in multiple population-based studies (70, 71). We also noted an association between lower maternal education and greater prevalence of anxiety at age 15, but this did not persist when adjusted for maternal age. Studies have examined the psychophysiological pathways underlying this association which have suggested that exposure to chronic stressful events secondary to low SES and long-term alterations in physiological systems make individuals more vulnerable to anxiety, depression and aberrant hypothalamic-pituitary-adrenal (HPA) axis functioning (McEwen, 2000). Receipt of public health insurance was additionally used as an estimate of socioeconomic disadvantage in the ELGAN cohort. Similar to our results, other longitudinal studies involving youth exposed to poverty and socioeconomic disadvantage were noted to have increased prevalence of symptoms of anxiety and depression in adolescence (72, 73).

Parental mental health plays a critical role in the child's physiological as well as psychological response to stress (71). Exposure to childhood trauma can lead to altered emotional and behavioral response patterns that often persist into adulthood and thus negatively impact caregiving (36, 74). Maternal report of a history of childhood trauma has been linked to mood and anxiety disorders, posttraumatic stress disorder, and other significant mental health conditions in their children (75–78). This critical public health implication was noted in our studied sample as well, as mothers who were exposed to traumatic events in their childhood were more likely to have children diagnosed with anxiety and depression in adolescence. Specifically, the association between mothers exposed to parental upheaval, sexual trauma or being victims of any other traumatic event, and the presence of anxiety and depressive symptoms in the adolescent was significant in both biological sexes. Maternal social isolation, dysfunction in intimate relationships, academic and social challenges may occur as downstream effects of a traumatic exposure in childhood and can impact access to healthcare and other resources for the child and family (35, 79–81). Surprisingly, the severity of childhood trauma experienced by the mother did not significantly alter the associations between perinatal factors and socioeconomic status and anxiety and/or depression outcomes. In our sample, it is important to consider the role of extreme preterm birth, the associated stressors related to medical investigations, interventions and monitoring, the child's co-occurring medical diagnoses and any signs of distress and helplessness that they may display, which could serve as traumatic reminders for mothers who report having experienced childhood trauma (82). This could lead to an added increased risk of insensitive caregiving, child maltreatment and long-term impact on mother-child relationships and maladaptive interaction styles, in part due to the mother's own impaired self-regulation abilities (34, 51, 82, 83).

## 5 Strengths and limitations

In addition to its prospective, longitudinal design with recruitment of a large number of children who were born extremely preterm, our study is unique in the use of a structured diagnostic interview to evaluate symptoms of anxiety and depression in adolescents at age 15. Information about maternal health was obtained by self-report leading to concerns about inaccurate reporting and the resultant misclassification bias could lead to an underestimation of the strength of associations with those health disorders (84). We have previously reported that in the ELGAN cohort some adolescents who had anxiety and depression symptoms on dimensional measures did not meet the clinical threshold for the diagnosis on the MINI-KID. This suggests that the interview does not fully capture subclinical symptoms in our study population (4). Another limitation of our study is the relatively high attrition rate at age 15 years, similar to that noted in other longitudinal studies (85). It is also important to consider the impact of our selected study population of extreme preterm infants where effects of maternal factors on the development of anxiety and depression may be underestimated since the study design controls for gestational age.

In addition, we do not have information on parental attachment styles in our analysis, making it difficult to assess the extent of emotional dysregulation's impact on anxiety and depression compared to other presented factors. A methodologic concern with including parental psychopathology as a confounder in our analyses is that ascertainment of parent psychiatric diagnoses was completed simultaneous with ascertainment of child's psychiatric diagnoses. It is possible that any symptoms of parental psychopathology reported at age 15 may not have been present at birth for all participants and may have arisen due to associated stressors related to EP birth, associated medical and psychiatric comorbidities in the child and accurate date regarding the timeline of the onset of symptoms in the parent was not available. Only data on maternal psychiatric diagnoses was available for a subset of the sample and we did not have paternal psychiatric diagnostic information on all youth. Similarly, our inclusion of maternal report of early life trauma obtained when adolescents were 15 years of age is not in temporal alignment with our other early life indicators. While we believe this variable is an important consideration in examining the psychiatric status of our adolescent sample, the ascertainment of this information in a retrospective manner may have biased our data in an unknown fashion, and follow-up of the influence of this variable for this population of adolescents remains a future area of scientific inquiry into modifiable maternal factors.

## 6 Conclusions

The current study underscores the importance of monitoring for psychiatric disorders, especially anxiety and depression, in children born extremely preterm. Maternal health conditions including exposure to smoke and elevated BMI were noted to be associated with the development of anxiety and depression in their child in adolescence. Additionally, socioeconomic stressors and maternal childhood trauma were independently associated with symptoms of anxiety and depression in these youth born extremely preterm. Identification of these modifiable risk factors serves to inform future research efforts and the design of interventions to effectively address these challenging and prevalent conditions in preterm children. These findings set the stage for further work aimed at identifying mediating pathways between maternal childhood traumatic exposures and social determinants of health and child mental health outcomes in adolescents who are vulnerable.

## Data Availability

The raw data supporting the conclusions of this article will be made available by the authors, without undue reservation.

## Project lead for ELGAN-2 & ELGAN ECHO: Julie V. Rollins, MA

### Site Principal Investigators

Baystate Medical Center, Springfield, MA: Bhavesh Shah, MD; Rachana Singh, MD, MS, Ruben Vaidya, MD.

Boston Children’s Hospital, Boston, MA: Linda Van Marter, MD, MPH and Camilla Martin, MD, MPH; Janice Ware, PhD, Caitlin Rollins, MD.

Tufts Medical Center, Boston, MA: Cynthia Cole, MD; Ellen Perrin, MD, Christina Sakai, MD.

University of Massachusetts Medical School, Worcester, MA: Frank Bednarek, MD (deceased); Jean Frazier, MD.

Yale University School of Medicine, New Haven, CT: Richard Ehrenkranz, MD (deceased); Jennifer Benjamin, MD, Angela Montgomery, MD.

Wake Forest University, Winston-Salem, NC: T Michael O’Shea, MD, MPH, Lisa Washburn, MD, Semsa Gogcu, MD, MPH.

University of North Carolina, Chapel Hill, NC: Carl Bose, MD; Diane Warner, MD, MPH, T Michael O’Shea, MD, MPH.

East Carolina University, Greenville, NC: Steve Engelke, MD, Amanda Higginson, MD, Jason Higginson, MD, Kelly Bear, MD.

Helen DeVos Children’s Hospital, Grand Rapids, MI: Mariel Poortenga, MD; Steve Pastyrnak, PhD.

Sparrow Hospital, Lansing, MI and Michigan State University, East Lansing, MI; Padu Karna, MD; Nigel Paneth, MD, MPH; Madeleine Lenski, MSPH.

University of Chicago Medical Center, Chicago, IL: Michael Schreiber, MD; Scott Hunter, PhD; Michael Msall, MD.

William Beaumont Hospital, Royal Oak, MI: Danny Batton, MD; Judith Klarr, MD, Young Ah Lee, MD, Rawad Obeid, MD.

### Site Study Coordinators

Baystate Medical Center, Springfield, MA: Karen Christianson, RN; Deborah Klein, BSN, RN, Katie Wagner, MS, Victoria Cobb, Shaula Paula, Andres Santana.

Boston Children’s Hospital, Boston MA: Maureen Pimental, BA; Collen Hallisey, BA; Taryn Coster, BA, Maddie Dolins, Maggie Mittleman, Hannah Haile, Julia Rohde, Kaysi Herrera Pujols, Susie Rodriquez, Kyla Waring.

Tufts Medical Center, Boston, MA: Ellen Nylen, RN; Emily Neger, MA; Kathryn Mattern, BA, Catherine Ma, Deanna Toner, Elizabeth Vitaro, Allison Nolan.

University of Massachusetts Medical School, Worcester, MA: Lauren Venuti, BA; Beth Powers, RN; Ann Foley, EdM, Taylor Merk, BA.

Yale University School of Medicine, New Haven, CT: Joanne Williams, RN; Elaine Romano, APRN, Christine Henry.

Wake Forest University, Winston-Salem, NC: Debbie Hiatt, BSN (deceased); Nancy Peters, RN; Patricia Brown, RN; Emily Ansusinha, BA, Jazmyne James, MS, Nou Yang, MS, Nicole Froelich, Kristi Lanier.

University of North Carolina, Chapel Hill, NC: Gennie Bose, RN; Janice Wereszczak, MSN; Janice Bernhardt, MS, RN.

East Carolina University, Greenville, NC: Joan Adams (deceased); Donna Wilson, BA, BSW, Nancy Darden-Saad, BS, RN, Bree Williams, Emily Jones, Hannah Morris, Taiara Williams, Isabella Carter, Emily Jones.

Helen DeVos Children’s Hospital, Grand Rapids, MI: Dinah Sutton, RN; Julie Rathbun, BSW, BSN, Stephanie Fagerman, William Boshoven, Jalen Johnson, Brandon James; Cynthia Gile, BS, CCRC, Megan Maynard, Emina Nakic, Cynthia Gile, Duvonna Haynes.

Sparrow Hospital, Lansing, MI and Michigan State University, East Lansing, MI: Karen Miras, RN, BSN; Carolyn Solomon, RN, Deborah Weiland, MSN, Chloe Caltrider.

University of Chicago Medical Center, Chicago, IL: Grace Yoon, RN; Rugile Ramoskaite, BA; Suzanne Wiggins, MA; Krissy Washington, MA; Ryan Martin, MA; Barbara Prendergast, BSN, RN, Emma Lynch, MPH, Sabina Hajdarovic.

William Beaumont Hospital, Royal Oak, MI: Beth Kring, RN.

### Other Study Personnel

Baystate Medical Center, Springfield, MA: Anne Smith, PhD; Susan McQuiston, PhD.

Boston Children’s Hospital: Samantha Butler, PhD; Rachel Wilson, PhD; Kirsten McGhee, PhD; Patricia Lee, PhD; Aimee Asgarian, PhD; Anjali Sadhwani, PhD; Brandi Henson, PsyD.

Tufts Medical Center, Boston MA: Cecelia Keller, PT, MHA; Jenifer Walkowiak, PhD; Susan Barron, PhD.

University of Massachusetts Medical School, Worcester MA: Alice Miller, PT, MS; Brian Dessureau, PhD; Molly Wood, PhD; Jill Damon-Minow, PhD.

Yale University School of Medicine, New Haven, CT: Elaine Romano, MSN; Linda Mayes, MD; Kathy Tsatsanis, PhD; Katarzyna Chawarska, PhD; Sophy Kim, PhD; Susan Dieterich, PhD; Karen Bearrs, PhD.

Wake Forest University Baptist Medical Center, Winston-Salem NC: Ellen Waldrep, MA; Jackie Friedman, PhD; Gail Hounshell, PhD; Debbie Allred, PhD.

University Health Systems of Eastern Carolina, Greenville, NC: Rebecca Helms, PhD; Lynn Whitley, PhD Gary Stainback, PhD.

University of North Carolina at Chapel Hill, NC: Lisa Bostic, OTR/L; Amanda Jacobson, PT; Joni McKeeman, PhD; Echo Meyer, PhD.

Helen DeVos Children’s Hospital, Grand Rapids, MI: Steve Pastyrnak, PhD.

Sparrow Hospital, Lansing, MI and Michigan State University, East Lansing, MI: Joan Price, EdS; Megan Lloyd, MA, EdS.

University of Chicago Medical Center, Chicago, IL: Susan Plesha-Troyke, OT; Megan Scott, PhD.

William Beaumont Hospital, Royal Oak, MI: Katherine M Solomon, PhD; Kara Brooklier, PhD; Kelly Vogt, PhD.

We would like to thank David Sheehan of the University of South Florida College of Medicine, for his consultation regarding the use of the Mini International Neuropsychiatric Interview for Children and Adolescents (MINI-KID).
