# Supplementary material for: Maternal and psychosocial antecedents of anxiety and depression in extremely low gestational age newborns at age 15 years
Source: Front Child Adolesc Psychiatry. 2024 Sep 13;3:1334316. doi: 10.3389/frcha.2024.1334316 (PMC11731609; doi:10.3389/frcha.2024.1334316)
Supplement: Supplementary file 1 [file Datasheet1.docx]

**Supplemental Table 1. Covariates use in multivariable analyses.**

| exposure | Covariates appropriate for Adjustment in multivariable analyses |
| --- | --- |
| Maternal Smoke exposure | Medicaid, mother education, marital status, **mother childhood trauma** |
| Maternal obesity | Medicaid, Mother education, **mother childhood trauma** |
| Maternal hypertension | Medicaid, mother education, mother obesity, mother smoke, **mother childhood trauma** |
| Maternal asthma | Medicaid, mother education, mother obesity, mother smoking, **mother childhood trauma** |
| Maternal diabetes | Medicaid, mother education, mother obesity, **mother childhood trauma** |
| Marital status | **Mother childhood trauma** |
| Mother education | Mother age, **mother childhood trauma** |
| Medicaid | Marital status, mother age, mother education, **mother childhood trauma** |
| CTQ | *No variables require adjustment* |

**Supplemental Table 2a:** Maternal health characteristics at birth: Univariate and Multivariate analyses with **Female** adolescent anxiety or depression.

| **Characteristic** | **Anxiety** | | **Depression** | | **Anx. OR Dep.** | | **Anx. AND Dep.** | |
| --- | --- | --- | --- | --- | --- | --- | --- | --- |
|  | **OR** | **95% CI** | **OR** | **95% CI** | **OR** | **95% CI** | **OR** | **95% CI** |
| **Univariable Analysis** |  |  |  |  |  |  |  |  |
| **Pre-pregnancy asthma** | 1.03 | 0.49, 2.09 | 1.16 | 0.50, 2.49 | 0.76 | 0.36, 1.53 | 1.91 | 0.80, 4.19 |
| **Pre-pregnancy diabetes** | 1.21 | 0.32, 3.95 | 1.32 | 0.29, 4.58 | 0.91 | 0.24, 2.97 | 2.05 | 0.44, 7.20 |
| **Pre-pregnancy high blood pressure** | 2.01 | 0.74, 5.26 | 1.13 | 0.31, 3.28 | 1.5 | 0.56, 3.92 | 1.77 | 0.48, 5.21 |
| **Pregnancy-induced hypertension** | 1.63 | 0.87, 3.01 | 1.06 | 0.49, 2.13 | 1.42 | 0.77, 2.59 | 1.3 | 0.56, 2.79 |
| **Preeclampsia** | 1.44 | 0.73, 2.79 | 0.85 | 0.35, 1.85 | 1.17 | 0.60, 2.24 | 1.14 | 0.44, 2.61 |
| **Discharge diagnosis of Hypertensive disorder** | 1.71 | 0.95, 3.02 | 1.61 | 0.84, 3.00 | **1.81** | **1.03, 3.15** | 1.57 | 0.74, 3.16 |
| **Discharge diagnosis of Hypertensive disorder** | 1.16 | 0.40, 3.02 | 1.9 | 0.65, 5.02 | 1.39 | 0.53, 3.55 | 1.67 | 0.46, 4.85 |
| **Hypertensive disorder during pregnancy** | 1.67 | 0.98, 2.84 | 1.42 | 0.77, 2.55 | 1.62 | 0.96, 2.70 | 1.55 | 0.77, 2.99 |
| **Prepreg BMI:** |  |  |  |  |  |  |  |  |
| Healthy Weight | — | — | — | — | — | — | — | — |
| Under Weight | 1.2 | 0.44, 3.00 | 1.08 | 0.29, 3.19 | 1.1 | 0.42, 2.67 | 1.3 | 0.28, 4.38 |
| Over Weight | 1.62 | 0.85, 3.05 | 1.74 | 0.82, 3.61 | 1.5 | 0.81, 2.77 | 2.19 | 0.94, 5.06 |
| Obese | **1.86** | **1.03, 3.38** | **2.57** | **1.33, 5.02** | **1.95** | **1.10, 3.46** | **2.9** | **1.36, 6.31** |
| **Smoke while pregnant** | 1.54 | 0.80, 2.89 | 1.76 | 0.86, 3.46 | 1.51 | 0.81, 2.82 | 1.97 | 0.89, 4.10 |
| **second-hand smoke exposure** | 1.54 | 0.90, 2.61 | **2.26** | **1.26, 4.00** | 1.5 | 0.89, 2.49 | **2.76** | **1.45, 5.24** |
| **Active or Second-hand Smoke Exposure** | 1.45 | 0.86, 2.42 | **2.12** | **1.20, 3.72** | 1.45 | 0.88, 2.37 | **2.48** | **1.31, 4.68** |
|  |  |  |  |  |  |  |  |  |
| **Multivariable Analysis** |  |  |  |  |  |  |  |  |
| **Pre-pregnancy asthma** *Adjusted for Medicaid, edu, obesity, smoke* | 0.91 | 0.41, 1.90 | 0.92 | 0.37, 2.09 | 0.67 | 0.30, 1.39 | 1.42 | 0.55, 3.37 |
| **Pre-pregnancy diabetes** *Adjusted for Medicaid, edu, obesity* | 0.86 | 0.22, 2.94 | 0.95 | 0.20, 3.46 | 0.67 | 0.17, 2.26 | 1.35 | 0.28, 5.13 |
| **Hypertension combined variable** *Adjusted for Medicaid, edu, obesity, smoke* | 1.51 | 0.84, 2.66 | 1.55 | 0.81, 2.91 | 1.6 | 0.92, 2.78 | 1.52 | 0.72, 3.09 |
| **Prepreg BMI:** *adjusted for Medicaid, edu* |  |  |  |  |  |  |  |  |
| Healthy Weight | — | — | — | — | — | — | — | — |
| Under Weight | 1.09 | 0.39, 2.82 | 0.98 | 0.26, 2.95 | 1 | 0.37, 2.48 | 1.16 | 0.25, 4.10 |
| Over Weight | 1.5 | 0.77, 2.87 | 1.59 | 0.74, 3.36 | 1.43 | 0.76, 2.67 | 1.89 | 0.79, 4.48 |
| Obese | 1.73 | 0.92, 3.25 | **2.44** | **1.23, 4.88** | **1.89** | **1.04, 3.44** | **2.58** | **1.17, 5.82** |
| **Smoke exposure combined** *Adjusted for Medicaid, edu, marital* | 1.18 | 0.61, 2.24 | **2.07** | **1.03, 4.21** | 1.11 | 0.60, 2.03 | **2.83** | **1.26, 6.48** |

OR = Odds Ratio, CI = Confidence Interval

Statistically significant associations are in bold.

**Supplemental Table 2b:** Maternal health characteristics at birth: univariate and multivariable analyses with **Male** adolescent anxiety or depression.

| **Characteristic** | **Anxiety** | | **Depression** | | **Anx. OR Dep.** | | **Anx. AND Dep.** | |
| --- | --- | --- | --- | --- | --- | --- | --- | --- |
|  | **OR** | **95% CI** | **OR** | **95% CI** | **OR** | **95% CI** | **OR** | **95% CI** |
| **Univariable Analysis** |  |  |  |  |  |  |  |  |
| **Pre-pregnancy asthma** | 1.31 | 0.58, 2.70 | 1.49 | 0.64, 3.19 | 1.45 | 0.73, 2.79 | 1.33 | 0.37, 3.75 |
| **Pre-pregnancy diabetes** | 1.37 | 0.20, 5.86 | 1.76 | 0.26, 7.56 | 2.49 | 0.60, 9.61 | 0 |  |
| **Pre-pregnancy high blood pressure** | 0.44 | 0.07, 1.54 | 1.76 | 0.56, 4.68 | 1.07 | 0.38, 2.68 | 0.62 | 0.03, 3.19 |
| **Pregnancy-induced hypertension** | 0.79 | 0.31, 1.76 | 1.25 | 0.51, 2.75 | 1.01 | 0.48, 1.99 | 0.93 | 0.21, 2.84 |
| **Preeclampsia** | 1.28 | 0.41, 3.34 | 1.66 | 0.53, 4.38 | 1.56 | 0.61, 3.70 | 1.3 | 0.20, 4.85 |
| **Discharge diagnosis of Hypertensive disorder** | 1.09 | 0.39, 2.60 | 1.38 | 0.49, 3.34 | 1.15 | 0.49, 2.51 | 1.49 | 0.34, 4.67 |
| **HELLP syndrome** | 0.48 | 0.03, 2.56 | 2.34 | 0.50, 8.41 | 1.14 | 0.25, 4.05 | 1.45 | 0.08, 8.08 |
| **Hypertensive disorder during pregnancy** | 0.99 | 0.45, 2.01 | 1.71 | 0.80, 3.43 | 1.28 | 0.67, 2.36 | 1.41 | 0.45, 3.71 |
| **Pre-pregnancy BMI:** |  |  |  |  |  |  |  |  |
| Healthy Weight | — | — | — | — | — | — | — | — |
| Under Weight | 1.83 | 0.56, 5.09 | 1.46 | 0.40, 4.30 | 2.32 | 0.87, 5.81 | 0.59 | 0.03, 3.19 |
| Over Weight | 2.06 | 0.98, 4.20 | 1.3 | 0.56, 2.85 | 1.88 | 0.97, 3.60 | 1.37 | 0.46, 3.64 |
| Obese | 1.76 | 0.86, 3.51 | 1.3 | 0.59, 2.72 | **2.13** | **1.16, 3.91** | 0.53 | 0.12, 1.72 |
| **Smoke while pregnant** | 1.23 | 0.53, 2.60 | 1.63 | 0.69, 3.52 | 1.27 | 0.61, 2.50 | 1.97 | 0.62, 5.24 |
| **Second-hand smoke exposure** | 0.48 | 0.19, 1.06 | 1.05 | 0.47, 2.16 | 0.82 | 0.42, 1.52 | 0.37 | 0.06, 1.30 |
| **Active or second-hand Smoke Exposure** | 0.77 | 0.37, 1.49 | 1.35 | 0.67, 2.60 | 0.97 | 0.53, 1.70 | 1.15 | 0.40, 2.87 |
|  |  |  |  |  |  |  |  |  |
| **Multivariable Analysis** |  |  |  |  |  |  |  |  |
| **Pre-pregnancy asthma** *Adjusted for Medicaid, edu, obesity, smoke* | 1.34 | 0.58, 2.92 | 1.37 | 0.57, 3.06 | 1.4 | 0.68, 2.78 | 1.45 | 0.37, 4.54 |
| **Pre-pregnancy diabetes** *Adjusted for Medicaid, edu, obesity* | 1.16 | 0.16, 5.50 | 1.48 | 0.20, 6.93 | 1.91 | 0.43, 7.90 | 0 |  |
| **Hypertension combined variable** *Adjusted for Medicaid, edu, obesity, smoke* | 0.89 | 0.39, 1.88 | 1.67 | 0.76, 3.49 | 1.21 | 0.62, 2.30 | 1.36 | 0.40, 3.94 |
| **Prepreg BMI:** *adjusted for Medicaid, edu* |  |  |  |  |  |  |  |  |
| Healthy Weight | — | — | — | — | — | — | — | — |
| Under Weight | 1.92 | 0.58, 5.50 | 1.5 | 0.40, 4.57 | 2.29 | 0.84, 5.84 | 0.83 | 0.04, 4.81 |
| Over Weight | **2.27** | **1.07, 4.75** | 1.39 | 0.59, 3.09 | **2.01** | **1.02, 3.90** | 1.33 | 0.42, 3.79 |
| Obese | 1.78 | 0.83, 3.75 | 1.37 | 0.61, 2.98 | **2.16** | **1.13, 4.11** | 0.56 | 0.12, 1.95 |
| **Smoke exposure combined** *Adjusted for Medicaid, edu, marital* | 0.76 | 0.32, 1.68 | 1.35 | 0.60, 2.99 | 0.76 | 0.38, 1.48 | 2.65 | 0.77, 8.40 |

OR = Odds Ratio, CI = Confidence Interval

Statistically significant associations are in bold.

**Supplemental Table 3a.** Maternal socioeconomic characteristics at birth: univariable and multivariate associations with **Female** adolescent anxiety or depression.

| **Characteristic** | **Anxiety** | | **Depression** | | **Anx. OR Dep.** | | **Anx. AND Dep.** | |
| --- | --- | --- | --- | --- | --- | --- | --- | --- |
|  | **OR** | **95% CI** | **OR** | **95% CI** | **OR** | **95% CI** | **OR** | **95% CI** |
| **Univariable Analysis** |  |  |  |  |  |  |  |  |
| **Marital Status** |  |  |  |  |  |  |  |  |
| Married | — | — | — | — | — | — | — | — |
| Separated/Divorced/  Widowed | **3.32** | **1.19, 9.68** | 1.93 | 0.58, 5.62 | 2.72 | 0.97, 7.90 | 2.6 | 0.78, 7.67 |
| Never married,  living together | 0.63 | 0.29, 1.26 | 1.28 | 0.61, 2.55 | 1.18 | 0.63, 2.16 | 0.44 | 0.13, 1.17 |
| Never married,  not living together | 1.34 | 0.66, 2.64 | 0.94 | 0.38, 2.09 | 1.33 | 0.67, 2.59 | 0.9 | 0.32, 2.18 |
| **Married** | 0.9 | 0.55, 1.48 | 0.82 | 0.47, 1.43 | 0.72 | 0.45, 1.15 | 1.18 | 0.62, 2.33 |
| **Education** |  |  |  |  |  |  |  |  |
| Less than High School | — | — | — | — | — | — | — | — |
| High School | 2.2 | 0.95, 5.46 | 1.97 | 0.80, 5.39 | 1.44 | 0.66, 3.20 | **6.27** | **1.70, 40.6** |
| Less than college | 1.3 | 0.54, 3.31 | 1.24 | 0.48, 3.51 | 0.84 | 0.37, 1.92 | 3.88 | 1.00, 25.7 |
| College | 1.07 | 0.43, 2.80 | 0.72 | 0.25, 2.19 | 0.78 | 0.34, 1.81 | 1.52 | 0.31, 11.0 |
| More than college | 1.22 | 0.48, 3.25 | 0.88 | 0.30, 2.68 | 0.85 | 0.36, 2.02 | 2.22 | 0.48, 15.7 |
| **Without College Degree** | 1.38 | 0.84, 2.31 | **1.86** | **1.04, 3.45** | 1.36 | 0.84, 2.21 | **2.26** | **1.13, 4.82** |
| **Insurance: Medicaid** | **1.71** | **1.04, 2.81** | 1.43 | 0.81, 2.48 | **1.69** | **1.05, 2.71** | 1.51 | 0.79, 2.82 |
| **Support: Food Stamps** | 1.57 | 0.79, 3.06 | 1.46 | 0.66, 3.01 | 1.62 | 0.83, 3.11 | 1.46 | 0.59, 3.25 |
|  |  |  |  |  |  |  |  |  |
| **Multivariable Analysis** |  |  |  |  |  |  |  |  |
| **Marital Status** |  |  |  |  |  |  |  |  |
| Married | — | — | — | — | — | — | — | — |
| Separated/Divorced/  Widowed | **3.32** | **1.19, 9.68** | 1.93 | 0.58, 5.62 | 2.72 | 0.97, 7.90 | 2.6 | 0.78, 7.67 |
| Never married,  living together | 0.63 | 0.29, 1.26 | 1.28 | 0.61, 2.55 | 1.18 | 0.63, 2.16 | 0.44 | 0.13, 1.17 |
| Never married,  not living together | 1.34 | 0.66, 2.64 | 0.94 | 0.38, 2.09 | 1.33 | 0.67, 2.59 | 0.9 | 0.32, 2.18 |
| **Education** *Adjusted for mat age* |  |  |  |  |  |  |  |  |
| Less than High School | — | — | — | — | — | — | — | — |
| High School | 2.19 | 0.93, 5.53 | 1.88 | 0.75, 5.24 | 1.36 | 0.61, 3.09 | **6.42** | **1.70, 42.1** |
| Less than college | 1.24 | 0.49, 3.35 | 1.13 | 0.41, 3.36 | 0.74 | 0.31, 1.78 | 4.01 | 0.97, 27.6 |
| College | 1.01 | 0.37, 2.85 | 0.64 | 0.20, 2.09 | 0.66 | 0.26, 1.66 | 1.57 | 0.30, 12.0 |
| More than college | 1.17 | 0.43, 3.36 | 0.79 | 0.25, 2.57 | 0.73 | 0.29, 1.88 | 2.31 | 0.47, 17.3 |
| **Insurance: Medicaid** *Adjusted for marital, age, edu* | 1.87 | 0.94, 3.78 | 1.22 | 0.58, 2.57 | 1.6 | 0.83, 3.10 | 1.53 | 0.66, 3.56 |

OR = Odds Ratio, CI = Confidence Interval. Statistically significant associations are in bold.

**Supplemental Table 3.b.** Maternal socioeconomic characteristics at birth: univariable and multivariate associations with **Male** adolescent anxiety or depression.

| **Characteristic** | **Anxiety** | | **Depression** | | **Anx. OR Dep.** | | **Anx. AND Dep.** | |
| --- | --- | --- | --- | --- | --- | --- | --- | --- |
|  | **OR***^1^* | **95% CI***^1^* | **OR***^1^* | **95% CI***^1^* | **OR***^1^* | **95% CI***^1^* | **OR***^1^* | **95% CI***^1^* |
| **Univariable Analysis** |  |  |  |  |  |  |  |  |
| **Marital Status** |  |  |  |  |  |  |  |  |
| Married | — | — | — | — | — | — | — | — |
| Separated/Divorced/  Widowed | 0.44 | 0.02, 2.41 | 0.66 | 0.04, 3.63 | 0.73 | 0.11, 2.96 | 0 |  |
| Never married,  living together | 0.63 | 0.25, 1.42 | 1.43 | 0.63, 3.06 | 1.21 | 0.61, 2.32 | 0.41 | 0.06, 1.46 |
| Never married,  not living together | 0.98 | 0.44, 2.04 | 1.12 | 0.46, 2.51 | 1.35 | 0.69, 2.58 | 0.42 | 0.06, 1.49 |
| **Married** | 1.32 | 0.73, 2.45 | 0.78 | 0.42, 1.46 | 0.79 | 0.48, 1.31 | 2.7 | 0.99, 9.49 |
| **Education** |  |  |  |  |  |  |  |  |
| Less than High School | — | — | — | — | — | — | — | — |
| High School | 0.48 | 0.18, 1.25 | 0.7 | 0.26, 1.87 | 0.54 | 0.24, 1.22 | 0.58 | 0.13, 2.58 |
| Less than college | 0.58 | 0.23, 1.46 | 0.41 | 0.13, 1.17 | 0.52 | 0.23, 1.18 | 0.28 | 0.04, 1.48 |
| College | 0.72 | 0.30, 1.77 | 0.93 | 0.38, 2.42 | 0.7 | 0.32, 1.54 | 1.15 | 0.34, 4.52 |
| More than college | 0.67 | 0.26, 1.73 | 0.51 | 0.17, 1.50 | 0.49 | 0.20, 1.15 | 0.91 | 0.23, 3.85 |
| **Without College Degree** | 0.9 | 0.51, 1.59 | 0.87 | 0.47, 1.60 | 1.04 | 0.63, 1.72 | 0.53 | 0.22, 1.24 |
| **Insurance: Medicaid** | 0.75 | 0.39, 1.37 | 1.06 | 0.54, 1.99 | 1.22 | 0.72, 2.03 | **0.19** | **0.03, 0.67** |
| **Support: Food Stamps** | 0.14 | 0.01, 0.68 | 1.14 | 0.37, 2.90 | 0.68 | 0.25, 1.61 | 0 |  |
|  |  |  |  |  |  |  |  |  |
| **Multivariable Analysis** |  |  |  |  |  |  |  |  |
| **Marital Status** |  |  |  |  |  |  |  |  |
| Married | — | — | — | — | — | — | — | — |
| Separated/Divorced/  Widowed | 0.44 | 0.02, 2.41 | 0.66 | 0.04, 3.63 | 0.73 | 0.11, 2.96 | 0 |  |
| Never married,  living together | 0.63 | 0.25, 1.42 | 1.43 | 0.63, 3.06 | 1.21 | 0.61, 2.32 | 0.41 | 0.06, 1.46 |
| Never married,  not living together | 0.98 | 0.44, 2.04 | 1.12 | 0.46, 2.51 | 1.35 | 0.69, 2.58 | 0.42 | 0.06, 1.49 |
| **Education** *Adjusted for mat age* |  |  |  |  |  |  |  |  |
| Less than High School | — | — | — | — | — | — | — | — |
| High School | 0.42 | 0.16, 1.14 | 0.67 | 0.25, 1.88 | 0.55 | 0.24, 1.29 | 0.36 | 0.08, 1.63 |
| Less than college | 0.48 | 0.18, 1.29 | 0.39 | 0.12, 1.22 | 0.54 | 0.23, 1.29 | **0.15** | **0.02, 0.83** |
| College | 0.59 | 0.23, 1.58 | 0.88 | 0.32, 2.55 | 0.73 | 0.31, 1.76 | 0.55 | 0.15, 2.25 |
| More than college | 0.56 | 0.20, 1.59 | 0.46 | 0.14, 1.52 | 0.52 | 0.20, 1.35 | 0.39 | 0.09, 1.78 |
| **Insurance: Medicaid** *Adjusted for marital, age, edu* | 0.62 | 0.24, 1.54 | 0.84 | 0.32, 2.20 | 0.95 | 0.44, 2.04 | 0.14 | 0.01, 0.84 |

OR = Odds Ratio, CI = Confidence Interval. Statistically significant associations are in bold.

**Supplemental Fig 1:** Directed acyclic graphs, DAGs, to determine a minimally sufficient set of adjustment variables for inclusion in the model for maternal pre-pregnancy weight.**
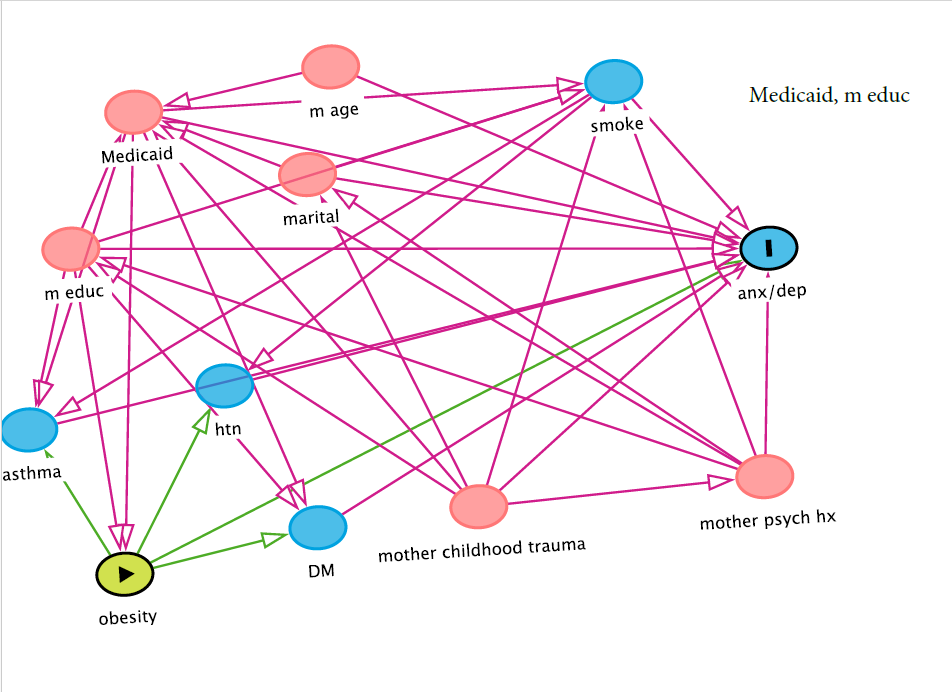
**

Abbreviations:
obesity: maternal pre-pregnancy BMI, educ: maternal education, DM: diabetes mellitus, mother psych hx: maternal psychiatric history, htn: maternal hypertension, m age: maternal age, marital: mother’s marital status

**Supplemental Fig 2:** Directed acyclic graphs, DAGs, to determine a minimally sufficient set of adjustment variables for inclusion in the model for maternal hypertension **
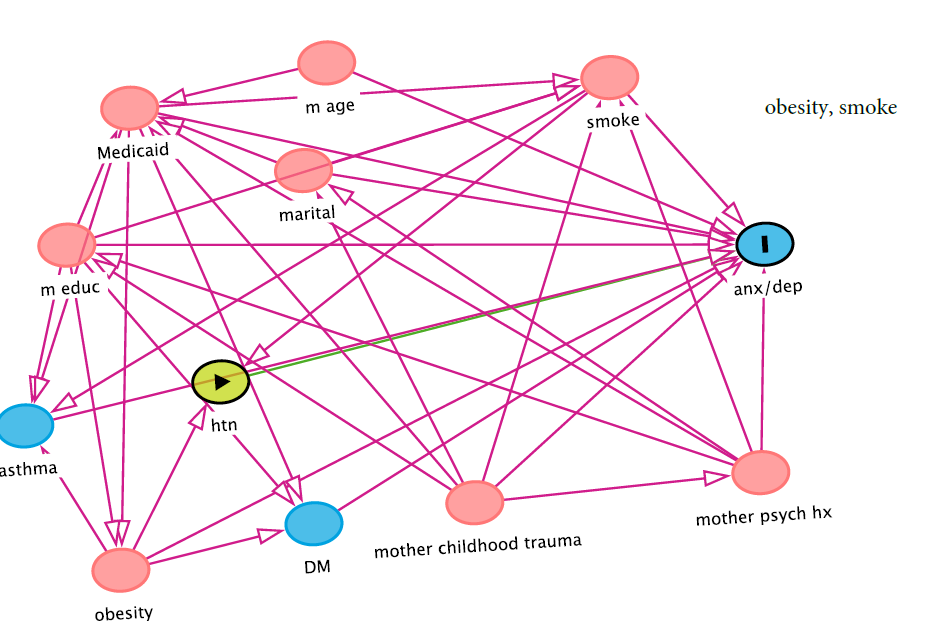
**

Abbreviations:
obesity: maternal pre-pregnancy BMI, educ: maternal education, DM: diabetes mellitus, mother psych hx: maternal psychiatric history, htn: maternal hypertension, m age: maternal age, marital: mother’s marital status, anx/dep: outcomes of anxiety and/or depression

**Supplemental Fig 3:** Directed acyclic graphs, DAGs, to determine a minimally sufficient set of adjustment variables for inclusion in the model for maternal asthma**
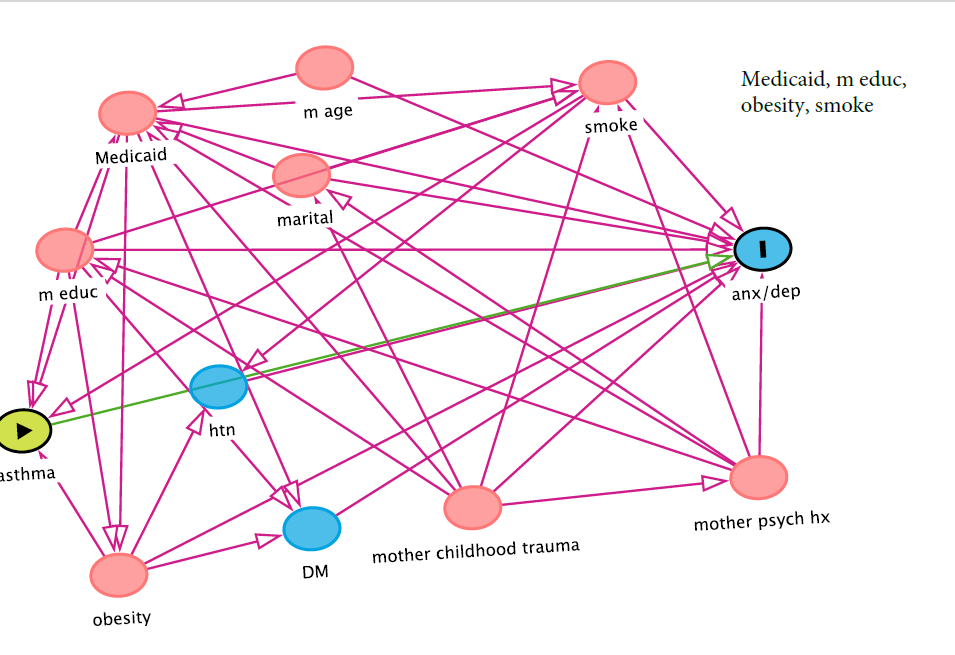
**

Abbreviations:
obesity: maternal pre-pregnancy BMI, educ: maternal education, DM: diabetes mellitus, mother psych hx: maternal psychiatric history, htn: maternal hypertension, m age: maternal age, marital: mother’s marital status, anx/dep: outcomes of anxiety and/or depression

**Supplemental Fig 3:** Directed acyclic graphs, DAGs, to determine a minimally sufficient set of adjustment variables for inclusion in the model for maternal smoking (active and second-hand).

**
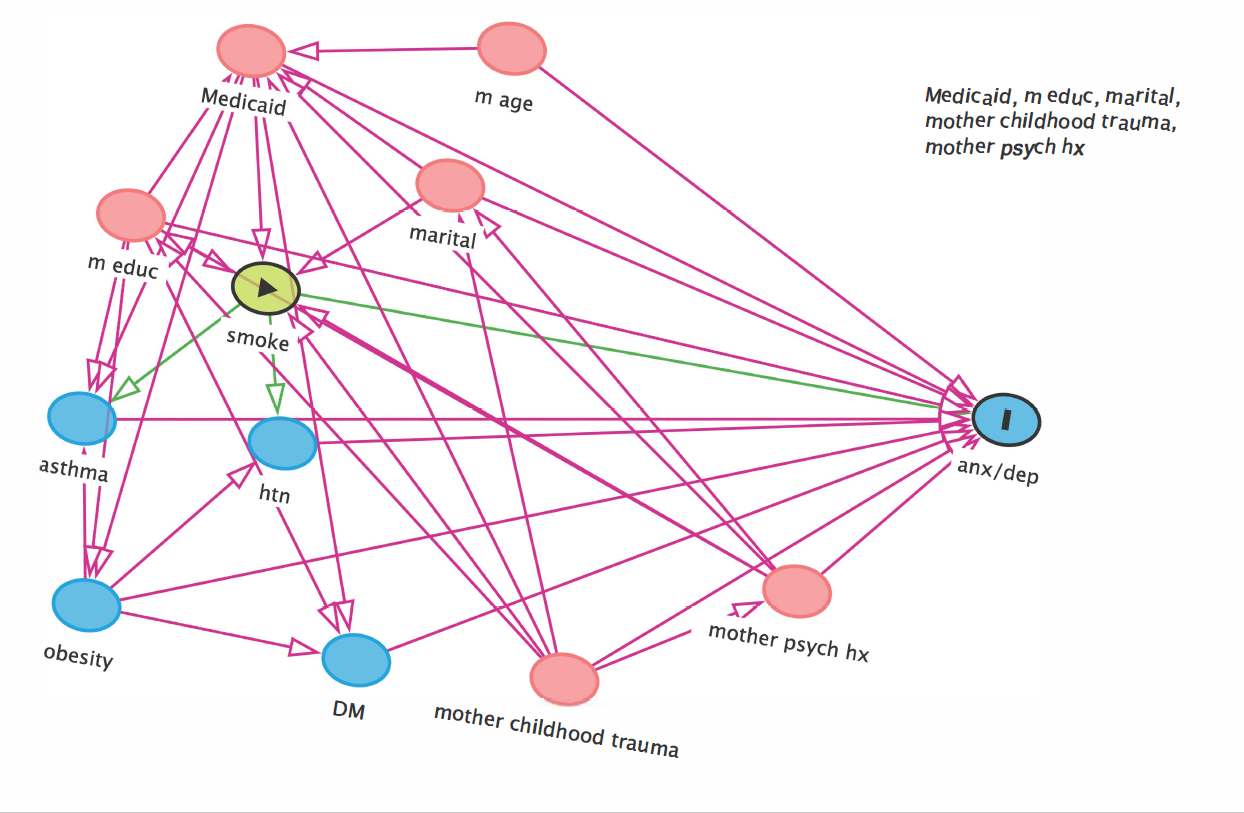
**

Abbreviations:
obesity: maternal pre-pregnancy BMI, educ: maternal education, DM: diabetes mellitus, mother psych hx: maternal psychiatric history, htn: maternal hypertension, m age: maternal age, marital: mother’s marital status, anx/dep: outcomes of anxiety and/or depression
